# Sequencing the exons of human glucocorticoid receptor (*NR3C1*) gene in Han Chinese with high-altitude pulmonary edema

**DOI:** 10.1186/s40101-018-0168-8

**Published:** 2018-03-27

**Authors:** Hui Du, Jing Zhao, Zhanhai Su, Yongnian Liu, Yingzhong Yang

**Affiliations:** 1 Department of Basic Medical Sciences, Medical College, Qinghai University, Xining, 810001 Qinghai China; 2 Research Center for High Altitude Medical Sciences, Medical College, Qinghai University, Xining, 810001 Qinghai China

**Keywords:** *NR3C1*, Exon, SNP, HAPE, Susceptibility

## Abstract

**Background:**

High-altitude pulmonary edema (HAPE) is a serious acute mountain sickness that mainly occurs in non-acclimatized individuals after rapid ascent to high altitude. The precise etiology of HAPE remains unclear. This study aimed to investigate whether *NR3C1* gene polymorphism is associated with the susceptibility to HAPE.

**Methods:**

The exons of *NR3C1* gene were sequenced by a ABI 3730 DNA analyzer in 133 HAPE patients and matched 135 healthy Han Chinese controls from the Yushu area in Qinghai (the altitude greater than 3500 m).

**Results:**

DNA sequencing showed the heterozygous substitutions at codon 588 (rs6194) in exon 6 and 766 (rs6196) in exon 9 of *NR3C1* gene. The genotypic distributions and allelic frequencies of *NR3C1* SNP rs6194 showed significant differences in two groups (*P* < 0.05). The frequencies of the C allele were significantly higher in the HAPE group than in the control group (*P* < 0.05) with an odds ratio of 3.009 (95% CI = 1.250-7.244). There were no differences in genotypic and allelic frequencies in rs6196 polymorphism between the two groups.

**Conclusions:**

*NR3C1* gene rs6194 polymorphism is correlated with HAPE susceptibility. CC genotype and C allele of rs6194 polymorphism might increase the risk of HAPE in Han Chinese.

## Background

High-altitude pulmonary edema (HAPE) is noncardiogenic pulmonary edema that usually occurs in rapidly ascending non-acclimatized individuals within the first week after arrival at altitudes above 2500 m [1, 2]. HAPE tends to be associated with exaggerated pulmonary hypertension and is defined as a non-inflammatory hemorrhagic pulmonary edema [3]. Circulating inflammatory markers are upregulated at high altitude, and HAPE patients show markedly elevated inflammatory markers in the bronchoalveolar lavage fluid. Thus, it is speculated that hypoxia-induced inflammation at high altitude may contribute to the development of HAPE [4, 5].

*NR3C1* (Nuclear Receptor Subfamily 3, Group C, Member 1) gene is located on chromosome 5q31-q32 and encodes human glucocorticoid receptor (GR). Glucocorticoids (GCs) bind to GR and translocate to the nucleus where they interact with glucocorticoid-responsive elements of different genes and regulate transcription. GCs inhibit the production of pro-inflammatory cytokines and stimulate the production of anti-inflammatory cytokines [6]. Variants in *NR3C1* gene may contribute to the spectrum of GC responses in different diseases [7]. Therefore, we performed a genetic sequencing and screening in HAPE patients to investigate possible association of genetic variations of *NR3C1* gene with HAPE.

## Methods

### Subjects

HAPE patients (HAPE) had been hospitalized during 2010 to 2017 in Yushu city (3760 m) of Qinghai province. The patients were diagnosed with HAPE based on the diagnostic criteria [8]. Healthy controls (Non-HAPE) were enrolled from the same area, with matched age, gender, and workplace (Table 1). These subjects had not suffered from HAPE or any other mountain sickness after reached at least 3 months. One hundred thirty-three HAPE and 135 matched Non-HAPE were enrolled. This study was approved by the Ethics Committee of the Medical College of Qinghai University, and every subject signed written consent. All subjects were of Han Chinese ethnicity and had no blood relationship with any other enrolled subject.

**Table 1:** Comparison of baseline data of two groups

Group	*n*	Gender	Age (years)	HGB (g/dL)	Hct (%)	HR (b/m)	SaO_2_ (%)
HAPE-p	133	Male	40.20 ± 9.91	157.24 ± 15.24*	47.79 ± 4.97*	109.73 ± 14.85*	62.46 ± 11.89*
HAPE-r	135	Male	40.92 ± 5.15	172.80 ± 14.54	50.74 ± 8.15	80.84 ± 12.03	88.85 ± 4.17

Values were means ± S.D.

*HAPE-p* high-altitude pulmonary edema patients, *HAPE-r* high-altitude pulmonary edema resistant (Control), *HGB* hemoglobin, *Hct* hematocrit, *HR* heart rate, *SaO*_*2*_ oxyhemoglobin saturation

*P < 0.05 vs. HAPE-r

### Sample collection and DNA extraction

Five milliliters of venous blood samples was collected from each participant. Blood samples were anticoagulated by EDTA. Genomic DNA was extracted from blood by Gentra Puregene Blood Kit (Qiagen, 158389, Germany) according to standard procedures. DNA samples were stored at − 20 °C until use.

### Exons PCR and sequencing

The primers of *NR3C1* gene (NG_009062) were designed by Primer3 (version 0.4.0) to encompass each exon except Exon 1. Exon 2 was divided into two parts due to its length (Table 2). The exons were amplified by PCR using Phusion® High-Fidelity PCR Master Mix (F-532L, Thermo Fisher Scientific Inc.). PCR cycles consisted of denaturation at 96 °C for 1 min, 30 cycles of 96 °C for 10 s, 60 °C for 30 s, and 72 °C for 30 s, and a final elongation at 72 °C for 5 min. Amplified fragments were resolved on 1.5% agarose gels stained with ethidium bromide. PCR products were bidirectionally sequenced with a ABI 3730 DNA analyzer by Shanghai Generay Biotech Co., Ltd, and compared with the published sequence of *NR3C1* gene.

**Table 2:** Amplification primers for *NR3C1* gene exons

Exon	Primer	Sequence	Tm (°C)	Length (bp)
2-1	GR-EXON2-1F	5′-TCGGATCAGGAAGATAATGTGA-3′	60	728
	GR-EXON2-1R	5′-GATCTCCAAGGACTCTCATTCG-3′		
2-2	GR-EXON2-2F	5′-AGACCAAAGCACCTTTGACATT-3′	60	773
	GR-EXON2-2R	5′-TTCCTACTTTCAAAAGGCCACT-3′		
3	GR-EXON3-F	5′-GCACTTGAAGCCAGAGTTCAC-3′	60	342
	GR-EXON3-R	5′-CACCCTGAGAAATGAAAACCA-3′		
4	GR-EXON4-F	5′-CAATACCTGTGGGTGTCTTGG-3′	60	387
	GR-EXON4-R	5′-TTCCCATTTTTATTGGGCAGT-3′		
5	GR-EXON5-F	5′-TTGAATAAACTGTGTAGCGCAGA-3′	60	501
	GR-EXON5-R	5′-CACCTGTATTCACCTGACTCTCC-3′		
6	GR-EXON6-F	5′-GACAGGGCTAATTGATCTCATTG-3′	60	307
	GR-EXON6-R	5′-ATCAGGAAAACATCAGCTGGTTA-3′		
7	GR-EXON7-F	5′-TTGCAAAACAAAACAAAAATGTG-3′	60	384
	GR-EXON7-R	5′-GGTGTCACTTACTGTGCCTTTCT-3′		
8	GR-EXON8-F	5′-GGATGACACAGTGAGACCCTATC-3′	60	391
	GR-EXON8-R	5′-TTGAACTCAAGCTATCACCAACA-3′		
9α	GR-EXON9α-F	5′-GGGAATTCCAGTGAGATTGGT-3′	60	459
	GR-EXON9α-R	5′-AACTGCTTCTGTTGCCAAGTC-3′		
9β	GR-EXON9β-F	5′-GTGTAACCCGGCTGGATAAAT-3′	60	508
	GR-EXON9β-R	5′-TCTGCTTTCAAACAGCACCA-3′		

*Tm* melting temperature

### Statistical analysis

SPSS software (version 17.0; SPSS, Inc, Chicago, IL, USA) was used for statistical analysis and *P* < 0.05 was considered as statistically significant. Hardy-Weinberg equilibrium (HWE) was used to assess the representativeness of the participants. Allele frequencies were calculated based on genotype frequencies in HAPE and Non-HAPE groups, and the intergroup difference was estimated with chi-square test. Odds ratios (ORs) and 95% confidence intervals (95% CIs) were used to evaluate the association between the variants and HAPE.

## Results

**Fig. 1:**
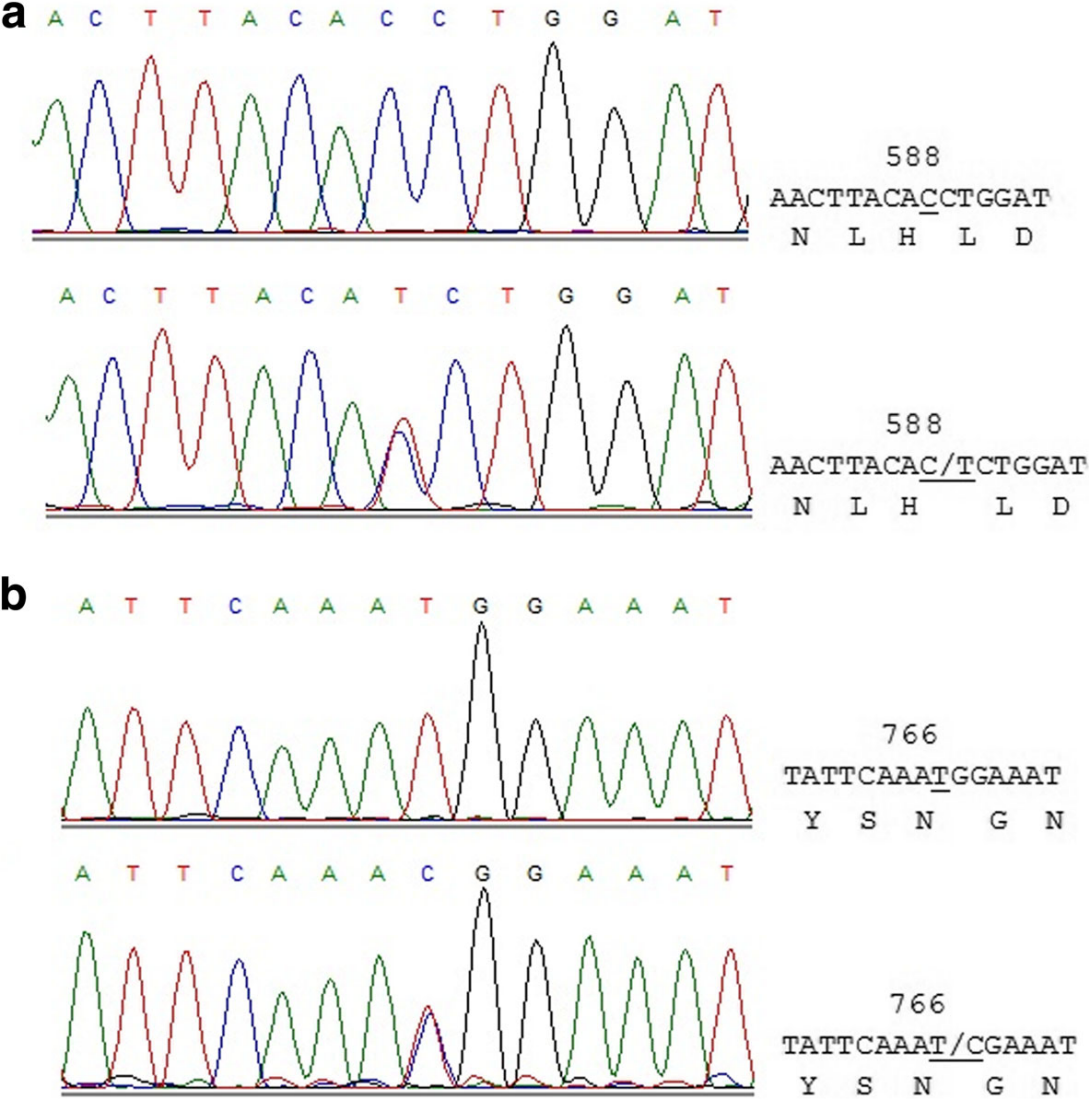
DNA sequencing results. **a** Partial nucleotide sequence of exon 6 in the region surrounding codon 588 (rs6194). **b** Partial nucleotide sequence of exon 9 in the region surrounding codon 766 (rs6196)

We sequenced the exons 2 to 9 of *NR3C1* gene in HAPE and Non-HAPE groups. DNA sequencing showed two single nucleotide substitutions at codon 588 in exon 6 (Fig. 1a) and codon 766 in exon 9 (Fig. 1b). We did not detect any differences in other exons. The variant in exon 6 was a silent C to T heterozygous change (CAC to CAT, both encoding histidine, synonymous codon). The variant in exon 9 was also a silent T to C heterozygous change (AAT to AAC, both encoding asparagine, synonymous codon). These polymorphisms had been deposited in dbSNP with the accession number rs6194 and rs6196, respectively. Genotype distributions of rs6194 and rs6196 polymorphisms did not deviate from HWE in HAPE and Non-HAPE groups (*P* = 0.754 for rs6194 in HAPE group, *P* = 0.346 for rs6194 in Non-HAPE group, *P* = 0.491 for rs6196 in HAPE group, and *P* = 0.216 for rs6196 in Non-HAPE group).

Frequency distributions of rs6194 polymorphism in HAPE group were different from Non-HAPE group (Table 3). CC genotype was more frequently observed in HAPE group than in Non-HAPE group, indicating a significant association with HAPE risk (*P* = 0.008, OR = 3.180, 95% CI = 1.299–7.825). C allele frequency showed a significantly increased trend in HAPE group than in Non-HAPE group (*P* < 0.05) with an odds ratio of 3.009 (95% CI = 1.250–7.244). There were no differences in genotypic and allelic frequencies in rs6196 polymorphism between the two groups (Table 4).

**Table 3:** Genotypic distributions and allelic frequencies of rs6194 in two groups

SNP	HAPE-p *n* = 132 (%)	HAPE-r *n* = 132 (%)	OR (95% CI)	*P* value
Genotype				
CC	125 (0.947)	112 (0.848)		
CT	7 (0.053)	20 (0.152)	3.180 (1.299–7.825)	0.008
TT	0 (0.00)	0 (0.00)		
Allele				
C	257 (0.973)	244 (0.924)	3.009 (1.250–7.244)	0.01
T	7 (0.027)	20 (0.076)		

*HAPE-p* high-altitude pulmonary edema patients, *HAPE-r* high-altitude pulmonary edema resistant, *OR* odds ratio

**Table 4:** Genotypic distributions and allelic frequencies of rs6196 in two groups

SNP	HAPE-p *n* = 133 (%)	HAPE-r *n* = 135 (%)	OR (95% CI)	*P* value
Genotype				
TT	118 (0.887)	109 (0.807)		
TC	15 (0.113)	26 (0.193)	1.876 (0.944–3.729)	0.07
CC	0 (0.00)	0 (0.00)		
Allele				
T	251 (0.944)	244 (0.904)	1.783 (0.922–3.448)	0.082
C	15 (0.056)	26 (0.096)		

*HAPE-p* high-altitude pulmonary edema patients, *HAPE-r* high-altitude pulmonary edema resistant, *OR* odds ratio

## Discussion

Circulating inflammatory markers are known to be upregulated at high altitude [9]. Serum levels of IL-6, IL-6 receptor, and C-reactive protein increased in healthy volunteers who spent three nights at an elevation higher than 3400 m [4]. The climbers at 8400 m had severe hypoxemia with subclinical HAPE [10]. Moreover, accumulation of inflammatory cells in multiple organs and elevated serum levels of cytokines were found in mice after short-term exposure to low oxygen concentrations [11–16]. These studies indicate that high altitude induces inflammation, which contributes to the development of HAPE. Anti-inflammatory agents are useful for prevention and treatment of HAPE [17].

Glucocorticoids regulate a broad spectrum of physiologic functions and play an important role in the regulation of inflammatory reactions. The effects of GCs are mediated by GR, which regulates the expression of glucocorticoid-responsive genes [6, 18]. Variations in *NR3C1* gene may contribute to GC responses, and a few rare genetic mutations in *NR3C1* have been found to substantially diminish GR function [7]. In the current study, we sequenced most exons of *NR3C1* gene and found heterozygous substitutions at codon 588 (rs6194) in exon 6 and 766 (rs6196) in exon 9 of *NR3C1* gene, both of them are the synonymous codon substitutions. The genotypic distributions and allelic frequencies of *NR3C1* SNP rs6194 were significantly different in two groups (*P* < 0.05). The frequencies of the C allele were significantly higher in the HAPE group than in the control group. There were no differences in genotypic and allelic frequencies in rs6196 polymorphism between the two groups. The rs6194 and rs6196 SNPs are located in the area that contains a ligand-binding domain (LBD), which is important for protein-protein interaction. The potential functional impacts of common SNPs in *NR3C1* gene, including rs6194 and rs6196, had been evaluated in COS-1 cells. Compared with wild-type, protein expression levels of GR variants (rs6194 C>T and rs6196 T>C) increased [19].

Several limitations of this study should be pointed out. First, we only sequenced the exons 2 to 9 and did not screen the promoter and exon 1. Second, the SNPs in intron and 5′ and 3′ UTR are valuable and should be genotyped in future studies. Third, a moderate number of participants were enrolled in the study.

## Conclusion

Our study indicates that *NR3C1* gene rs6194 polymorphism is correlated with HAPE susceptibility, and the CC genotype and C allele might increase the risk of HAPE in Han Chinese. Screening *NR3C1* gene polymorphism may help identify subjects susceptible to HAPE and guide them to take prevention measures.
